# Characterization of a metazoan ADA acetyltransferase complex

**DOI:** 10.1093/nar/gkz042

**Published:** 2019-01-31

**Authors:** Jelly H M Soffers, Xuanying Li, Anita Saraf, Christopher W Seidel, Laurence Florens, Michael P Washburn, Susan M Abmayr, Jerry L Workman

**Affiliations:** 1 Stowers Institute for Medical Research, Kansas City, MO 64110, USA; 2 Department of Pathology and Laboratory Medicine, University of Kansas Medical Center, Kansas City, KS 66160, USA; 3 Department of Anatomy and Cell Biology, University of Kansas School of Medicine, Kansas City, KS 66160, USA

## Abstract

The Gcn5 acetyltransferase functions in multiple acetyltransferase complexes in yeast and metazoans. Yeast Gcn5 is part of the large SAGA (Spt-Ada-Gcn5 acetyltransferase) complex and a smaller ADA acetyltransferase complex. In flies and mammals, Gcn5 (and its homolog pCAF) is part of various versions of the SAGA complex and another large acetyltransferase complex, ATAC (Ada2A containing acetyltransferase complex). However, a complex analogous to the small ADA complex in yeast has never been described in metazoans. Previous studies in *Drosophila* hinted at the existence of a small complex which contains Ada2b, a partner of Gcn5 in the SAGA complex. Here we have purified and characterized the composition of this complex and show that it is composed of Gcn5, Ada2b, Ada3 and Sgf29. Hence, we have named it the metazoan ‘ADA complex’. We demonstrate that the fly ADA complex has histone acetylation activity on histones and nucleosome substrates. Moreover, ChIP-Sequencing experiments identified Ada2b peaks that overlap with another SAGA subunit, Spt3, as well as Ada2b peaks that do not overlap with Spt3 suggesting that the ADA complex binds chromosomal sites independent of the larger SAGA complex.

## INTRODUCTION

The proper execution of chromatin-templated processes such as transcription requires distinct chromatin structures. Chromatin structure is dynamically regulated by large, multi-subunit chromatin-modifying complexes. Chromatin-modifying complexes can reposition nucleosomes and/or post-translationally modify histones and regulate chromatin accessibility. Multiple histone modifying complexes participate in the activation or repression of gene expression via various post-translational modifications of N-terminal histone tails. One major class of such histone modifications is post-translational lysine acetylation. Histone acetylation contributes to gene regulation by influencing the superhelical writhe constrained by the nucleosome (1,2). In addition, histone acetylation facilitates the interaction of transcription factors with nucleosomal DNA (3,4), and acetylated lysines can recruit effector proteins or readers that ‘dock’ onto these acetylated lysines to direct specific downstream events (reviewed in (5,6)).

Several protein complexes containing histone acetyl transferase (HAT) activity have been identified in yeast. Nucleosomal Acetyltransferase of histone H3 (NuA3) and NuA4 predominantly acetylate histones H3 and H4, respectively (7). Sas3 is the catalytic subunit of the NuA3 complex (8) whereas Esa1 is the catalytic subunit of NuA4 (9).

Gcn5 is another highly conserved acetyl transferase that is found in multiple HAT complexes (10–15). Recombinant Gcn5 as an individual protein acetylates histone H3 preferentially on lysine 14 (16). Though it is able to acetylate recombinant H3 lysine 14 *in-vitro* (17) incorporation of Gcn5 into multiprotein complexes potentiates its enzymatic activity (10,18–20). Gcn5 acetylates nucleosomal H2B and H3 as part of the HAT module of the 1.8 MDa Spt-Ada-Gcn5 acetyltransferase (SAGA) complex (10). The SAGA complex HAT module is composed of Gcn5, Ada2, Ada3 and Sgf29, all critical subunits for its activity and that endow expanded lysine specificity (21–26). Gcn5 acetylates residues H3K14>K3K18>H3K9 of a recombinant H3 peptide and nucleosomal substrates as part of the SAGA complex.

Interestingly, yeast Gcn5 was also found in a smaller 0.8 MDa ADA-containing complex. This ADA complex also contains Gcn5, Ada2, Ada3 and Sgf29, but in addition it contains ADA HAT component 1 (Ahc1) and Ahc2 (10,27,28). The ADA complex acetylates nucleosomal H2B and H3 with a potency equivalent to that of the SAGA complex (26). To date, a metazoan ortholog of this small ADA complex has not been characterized.

The *in-vivo* HAT-dependent transcriptional regulatory activities of Gcn5 depend on its interaction with Ada2 (29), a feature that is conserved in higher eukaryotes (12,30). Multiple forms of Ada2 exist in *Drosophila* and other higher eukaryotes (31,32). Both Ada2a and Ada2b are required for normal development but have distinct functions (14). Ada2a is specific to the ATAC complex (33), whereas Ada2b associates with Gcn5 in the SAGA complex (30,33–35). In Drosophila, alternative splicing produces two Ada2b isoforms, one long 62 kDa isoform referred to as Ada2b isoform B (Ada2b-PB), and a shorter 46 kDa isoform. Both isoforms share the N-terminal region, which is required for the interaction with Gcn5 (35). The short Ada2b-PA isoform associates with Gcn5, Ada3, Sgf29 and Chiffon forming the Chiffon Histone Acetyltransferase (CHAT) complex (36). Only the long Ada2b-PB isoform has been purified with the *Drosophila* SAGA complex (37). Thus, it appears to be the major if not the only form of Ada2B present in SAGA. Previous work supports the existence of a small Ada2b complex in *Drosophila*. For example, biochemical studies on SAGA detected a small Ada2b-containing complex by gel filtration (38). However, which Aa2b isoform forms this complex is not clear. Furthermore, the exact composition and function of this Ada2b-containing complex have not been characterized.

In this study, we biochemically purified the *Drosophila* small nuclear Ada2b complex and determined its composition via Multidimensional Protein Identification Technology (MudPIT). We demonstrated that it is composed of Ada2b-isoform B together with Gcn5, Ada3 and Sgf29 but lacks subunits homologous to yeast Ahc1/2. Hence, we have named it the metazoan ‘ADA complex’. We further show that this complex has histone acetylation activity on histones and nucleosome substrates. Moreover, ChIP-Sequencing experiments identified Ada2b peaks that overlap with another SAGA subunit, Spt3, as well as Ada2b peaks that do not overlap with Spt3 peaks suggesting that the ADA complex binds chromosomal sites independent of the larger SAGA complex. We therefore propose that the small nuclear Ada2b-PB complex represents a functional metazoan ADA complex.

## MATERIALS AND METHODS

### ChIP-Sequencing analysis

ChIP for factors Ada2b, Spt3, and Sgf11 in stage 4–6 embryos has been described (39). Data was aligned to *Drosophila* genome version dm3, using bowtie with parameters -k 1 -m 3. Gene definitions utilize Ensembl 78. Data is available via Gene Expression Omnibus accession number GSE98865.

Peaks were called using MACS2 under default parameters. Peaks present in at least two of three Spt3 or Sgf11 replicates, or at least three out of four Ada2b replicates, were combined and reduced to create a reference peak set for potential subunit binding locations. Reference peaks overlapping only Ada2b, and not Spt3 or Sgf11 are then defined as ada2b-only loci, whereas reference peaks overlapping all three factors (Ada2b, Sgf11, and Spt3), are considered sites of canonical SAGA binding. ChIP enrichment for each factor at each reference locus was calculated as the log2 ratio of the ChIP RPM signal over the total chromatin signal, and then averaged across replicates. *Z*-scores for the reference loci were calculated for each factor by subtracting the population mean from and dividing by the population standard deviation.

Intensity maps represent ChIP signal at TSS ±500 bp from averaged replicates in RPM and were constructed using R and the ComplexHeatmap package from Bioconductor (40).

### Fly strains and S2 cells

*Drosophila melanogaster* were grown on standard medium at 25°C. Embryos (0–12 h 25°C) were collected using standard apple juice plates with yeast. The Ada2b-isoform C-terminally tagged fly line w; P{w[+mC] = [UAS-ADA2B555-HA1FLAG2}, actin-Gal4/CyO, P{ry[+t7.2] = en1}wg[en11] was generated by recombining *w; P{w+mC = UAS-ADA2B-HA1FLAG2}* (41) *and w[1118], actin-Gal4/CyO*. Wild type refers to OregonR flies.

Stable S2 cell lines expressing Ada2b (isoform B; NP_001027151.1) in the pRmHa3-CHA2FL2 (Ada2b-PBH_2_F_2_) were maintained at 1–2 e^6^ cells/mL in SFX medium (HyQ SFX-Insect;HyClone Laboratories, Inc.) (37).

### Preparation of crude nuclear extract

Nuclear extract was prepared from fly embryos (0.5 g) or *Drosophila* S2 cells (sample from the large-scale culture described below). Extraction conditions were as previously described with the following modifications (31,41). S2 nuclei were extracted with high-salt buffer (20 mM HEPES, pH 7.4, 25% glycerol, 420 mM NaCl, 0.1% Triton X-100, 1 mM DTT, 1 mM PMSF, 0.2% [w/v] leupeptin, 0.2% [w/v] pepstatin A, 1× Roche cOmplete Protease Inhibitor Cocktail Tablets; Milipore Sigma, St Louis, MO USA) for one hour at 4°C. Nuclear proteins from fly embryo nuclei were extracted for one hour at 4°C in high salt buffer in (20 mM HEPES, pH 7.4, 10% glycerol, 420 mM NaCl, 1 mM MgCl_2_, 0.1% Triton X-100, 1 mM DTT, 1 mM PMSF, 0.2% [w/v] leupeptin, 0.2% [w/v] pepstatin A, 1× Roche cOmplete Protease Inhibitor Cocktail Tablets). Where low salt buffer is indicated, 420 mM NaCl was replaced with 150 mM NaCl. Extracts were treated with 25 U benzonase (Millipore Sigma, Burlington, MA, USA) per 10 mg and centrifuged for one hour at 50 000 rpm prior to gel filtration.

### Affinity purification

Ada2b complexes were Flag-affinity purified from 12 l of 1e^7^/l S2 Ada2b-PBH_2_F_2_ cells as described before with minor modifications to accommodate MudPIT analysis after gel filtration (31). The nuclear extract (∼800 mg) was treated with benzonase for 1 h at 4°C, followed by ultracentrifugation for two hours at 50 000 RPM at 4°C. The cleared nuclear extract was frozen and thawed on ice for a three hours affinity purification with M2 mouse-anti Flag conjugated beads (Millipore Sigma) at a ratio of 100 mg extract to 75 μl packed beads. After four high salt batch washes, Ada2b-PB complexes were eluted for one hour in one bead volume of 0.5 mg/ml 3× Flag peptide (Custom order, Penn State Milton S. Hershey Medical Center College of Medicine, NH2-END-Asp-Tyr-Lys-Asp-Asp-Asp-Asp-lys-Gly-Asp-Tyr-Lys-Asp-Asp-Asp-Asp-Lys-COOH) in 300 mM NaCl, 1.5 mM MgCl_2_, 5% glycerol, 0.05% Triton X-100, 20 mM HEPES pH 7.5 and 1 mM PMSF, 0.2% [w/v] leupeptin, 0.2% [w/v] pepstatin A, 1× Roche cOmplete Protease Inhibitor Cocktail Tablets at 4°C.

For HAT assays, the Flag immunoprecipitation was performed overnight, and the complexes were eluted for 1 h in 1.0 mg/ml 3× Flag in 150 mM NaCl, 1.5 mM MgCl_2_, 5% glycerol, 0.05% Triton X-100, 20 mM HEPES pH 7.5, 1 mM PMSF, 0.2% [w/v] leupeptin, 0.2% [w/v] pepstatin A, and 1× Roche cOmplete Protease Inhibitor Cocktail Tablets at 4°C.

### Gel filtration

A Superose 6 10/300 column (GE healthcare, Marlborough, MA, USA) was equilibrated with 300 mM NaCl, 1.5 mM MgCl_2_, 5% glycerol, 20mM HEPES pH 7.4. It was calibrated with the globular marker proteins aldolase thyroglobulin (Stokes radius (*R*_s_) = 8.5 nm), apoferritin (6.1 nm), aldolase (4.8 nm) ovalbumin (2.8 nm), carbonic anhydrase (2.1 nm), and dextran 2000 to determine the void volume (7.4 ml).

For nuclear extract, 1.5 mg total protein in 500 μl was injected. Up to 500 μl of Flag eluate was used for injection followed by HAT assays, and up to 1 ml eluate was pooled for gel filtration followed by MudPIT. Each sample was fractionated at 0.2 ml/min at 4°C and 500 μl fractions were collected. Per fraction, 20 μl was analysed by Western blot, with either 10–15 μg of input nuclear extract as input or 4–5 μl Flag purification eluate as input.

For repeat fractionation of Ada2b-PB containing complexes, an initial gel filtration of 1.5 mg nuclear extract from control S2 cells was performed to isolate the ∼1.8 kDa SAGA-complex containing fractions 9+10. Fractions 9+10 were combined to a volume of 1 ml, kept on ice, and injected without further concentration directly after the column was washed with one column volume buffer. The complete fractions column was TCA-precipitated, resolved in 20 μl 6 M urea and probed by Western blot.

### Western blotting analysis

The gel filtration samples were resolved on 10% SDS-PAGE gels. The HAT assay samples were resolved on 15% SDS-PAGE gels. Gels were transferred to a PVDF membrane and blocked for 1 h at 4°C in 5% milk in Tris-buffered saline (TBS) and 0.1% Tween-20. Primary antibodies were diluted in 5% milk in TBS and 0.1%Tween-20 and incubated overnight at 4°C. The following antibodies were used: Gcn5 (rabbit polyclonal, 1:1000, (GenScript antibody services, Atlanta, GA, USA anti full-length Gcn5); Ada2b (rabbit polyclonal, 1:1000; GenScript anti-amino-acid 1–330); Flag- horseradish peroxidase (mouse, 1:5000; Sigma Millipore); H3 (rabbit, 1:10 000; Abcam); H3K9/K14ac (rabbit, 1:1000, Sigma Millipore); H3K27ac (Rabbit, 1:1000, ab4729); beta-actin (mouse, 1:1000, ab 822); Donkey anti-rabbit IgG-horseradish peroxidase (1:5000, Fisher Scientific).

### MudPIT analysis

TCA-precipitated gel filtration fractions were prepared for MudPIT analysis as described before (42). Briefly, the samples were digested with endoproteinase LysC followed by trypsin, loaded onto microcapillary columns packed with three immobilized phases (reversed phase; strong cation exchange; reversed phase) and then eluted from the column using an Agilent 1100 or 1200 series quaternary HPLC pump. Peptides were resolved using 10 multidimensional chromatography steps (43) and analysed using a linear ion trap mass spectrometer (LTQ, Thermo Scientific).

### HAT assays

For all assays, input refers to the Flag-affinity-purified Ada2b-PB complexes and the fraction number indicates the gel filtration fraction containing the size-separated Ada2b-containing complex. Input (10ul) and alternating gel filtration fractions in the range of fraction 7–23 (22.5 μl) were incubated for 30′ at 30°C with 0.5 μg histone/nucleosome substrate in a 30 μl HAT reaction (0.05 mM Acetyl-CoA (Millipore Sigma) 225 mM NaCl, 5% glycerol, 1 mM DTT). Enzyme and/or Acetyl-CoA was omitted from the control reactions. Substrates were human recombinant H3.1 (New England Biolabs, Ipswich, MA, USA), acid-extracted total HeLa core histones separated by anion exchange (44) and HeLa polysomes (Epicypher, Durham, NC, USA).

## RESULTS

### Ada2b associates with chromatin independently of the canonical SAGA complex

**Figure 1.**
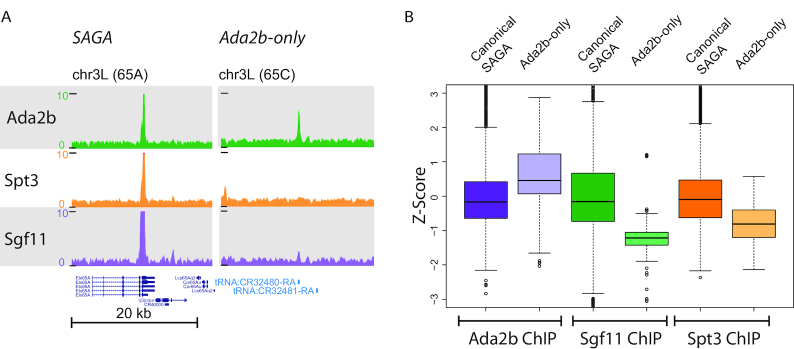
A subset of Ada2b binding sites do not correspond to canonical SAGA sites. (**A**) Genome browser screenshot shows genes bound by canonical SAGA (Ada2b, Spt3, Sgf11), and genes bound exclusively by Ada2b (note the absence of Spt3 and Sgf11 binding). (**B**) Box plots of the *Z*-scores of the canonical SAGA peaks and Ada2b-only peaks for subunits in the HAT, SPT and DUB modules of SAGA. Each boxplot shows the *Z*-score for either canonical SAGA or Ada2b-only loci. Canonical SAGA medians are centered around zero, indicating that their distribution is similar comparable to the *Z*-score distribution of the peak enrichments observed in the reference peak dataset. The distribution of the Ada2b signal for the Ada2b-only peaks is positively skewed, indicating that Ada2b is enriched at these sites. In contrast, the Sgf11 and Spt3 distribution was skewed to a value ≤-1, indicating that these factors are not enriched at the Ada2b-only peaks.

Previous studies exploring the modular nature of the SAGA histone acetyltransferase complex revealed that both enzymatic activities of this complex are not always required for gene regulation. Gene expression requirements for the histone acetyltransferase and histone de-ubiquitinase activity differed significantly during *Drosophila* oogenesis and embryogenesis (39). We previously examined the chromatin binding patterns of the SAGA-specific subunits genome wide by chromatin immunoprecipitation sequencing (ChIP-Seq) (39). Subunits Ada2b, Spt3 and Sgf11 were chosen as representative of the SAGA histone acetyltransferase (HAT), Suppressor of Ty (SPT) and deubiquitinase modules (DUB), respectively. A reference set of genomic binding locations was created by taking the union of peaks called by MACS for each factor. Only peaks reproducibly present among replicates were included in the set. As such, the reference peak data set contains a curated list of loci that most likely represents SAGA subunit binding sites. As was reported previously, the peak overlap identified 1650 canonical SAGA sites, and 2382 unique Sgf11 binding sites (39), Supplemental Figure S1A). Further experiments revealed that the DUB module binds to chromatin as both part of the SAGA complex and independent of SAGA (39). This data also revealed 267 sites occupied by Ada2b but lacking other SAGA subunits (‘Ada2b-only’ peaks) (Figure 1A, Supplemental Figure S1A), suggesting the possibility that the HAT module may also bind to chromatin independent of SAGA.

In further analysis of the Ada2b-only peaks, we compared subunit enrichment at the Ada2b-only bindings sites and at canonical SAGA binding sites. Since the absolute ChIP enrichment differed for each subunit (Supplemental Figure 2B), we examined the distribution of *Z*-scores for each factor at Ada2b-only binding sites versus canonical SAGA binding sites. At canonical SAGA sites, each factor had comparable *Z*-scores, indicating that the subunits have similar enrichment distributions at the canonical SAGA loci (Figure 1B). In contrast, at the Ada2b-only sites, *Z*-scores remained high for Ada2b, but were very low for Sgf11 and Spt3, suggesting differential enrichment for these factors at these locations, with Ada2b being enriched, but not Spt3 or Sgf11.

Therefore, we hypothesize that the Ada2b-only sites are a functionally distinct set of Ada2b-occupied genomic loci. The SAGA complex is known to be enriched at the TSS of its target genes (39). Could the Ada2b-only peaks reflect loci where Ada2b tended to colocalize but perhaps not exactly overlap with Spt3 and Sgf11 at the gene promoter, or were these genes also lacking canonical SAGA at the gene promoter? To investigate how the subunits were juxtaposed at the gene promoter, the Ada2b-only peaks were mapped to the closest TSS, which recovered 189 unique TSSs within 1 kb of an Ada2b-only peak. Next, the peak intensity signals (RPM) for Ada2b, Spt3 and Sgf11 were mapped relative to these TSSs. Previously reports indicated that Ada2b binds tRNA genes (41). Here, 50% of these Ada2b-only peak associated genes corresponded to tRNA genes, though not all tRNA genes had Ada2b peaks. These sites were not bound by other SAGA subunits, suggesting that they are ADA and not SAGA sites. The peak intensity map shows that Ada2b is enriched at these promoters, but not Sgf11 and Spt3 (Supplemental Figure S1B). For comparison, we also mapped the peak intensity signal for a sample of 1000 canonical SAGA-bound genes, where one can observe the overlap in Ada2b, Spt3 and Sgf11 signal centered at the TSS (Supplemental Figure S1C). Note that the RPM of Sgf11 is significantly stronger at canonical SAGA binding sites than the Sgf11 signal observed at the unique Ada2b sites, consistent with their higher enrichment scores (Supplemental Figure S1A). Therefore, the genes uniquely occupied by Ada2b seem not to be bound by the canonical SAGA complex, but instead bound by Ada2b in a SAGA-independent fashion.

As the DUB module subunit Sgf11 can associate with chromatin as part of SAGA or as part of the isolated DUB module, we cannot exclude that non-canonical Sgf11 signal overlaps with that of Ada2b as part of an isolated HAT module or a novel complex. Therefore, we asked to what extent Ada2b binding overlaps with Spt3 binding as representative subunit for the intact SAGA complex. A total of 42% of genomic Ada2b peaks did not show overlap with Spt3, indicating sites of Ada2b binding that are distinct from (or independent of) the canonical SAGA complex (Supplemental Figure S1A). Importantly, these sites are unlikely to reflect the genes regulated by the Gcn5 containing ATAC complex, since that complex contains the alternative Ada2a subunit and not Ada2b. To date, Ada2b has been identified only as a subunit of SAGA. Additionally, yeast Ada2 has been found in both SAGA and in a smaller ADA complex. Therefore, we wondered if these results implied that a complex analogous to the *S. cerevisiae* ADA exists in *Drosophila* that is capable of binding to chromatin.

### Ada2b is a component of a small nuclear protein complex

**Figure 2.**
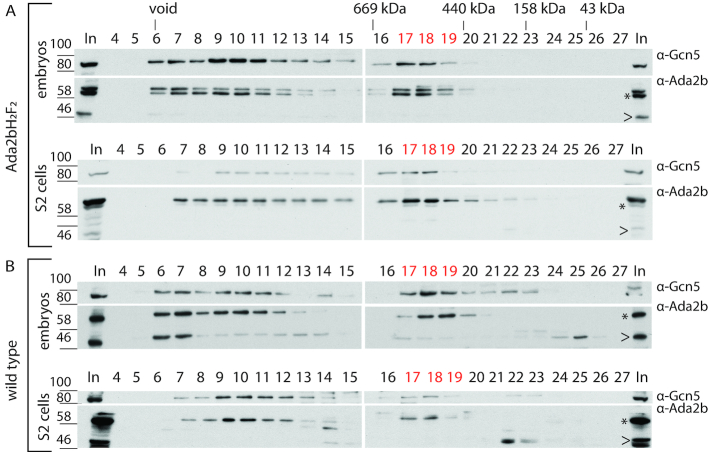
Ada2b-isoform B is present in a small nuclear protein complex. Nuclear extracts (1.5 mg) from embryos expressing Ada2b-PBH_1_F_2_ and S2 cells expressing Ada2b-PBH_2_F_2_ were size-fractionated by Superose 6 gel filtration. The fractionation profile for Gcn5 and Ada2b was determined by Western blot. (**A**) Gcn5 and Ada2b consistently co-eluted in high molecular weight fractions 9–12, as expected for SAGA. They also co-eluted in smaller molecular weight fractions 17–19. (**B**) Endogenous Ada2b-PB consistently eluted in fractions 17–19 in extracts from wild-type embryos and S2 cells, indicating that the small complex is not a consequence of tagged Ada2b-PB overexpression. Fraction numbers and marker sizes are indicated, In: input. Asterisk (*): 62 kDa Ada2b-isoform B (Ada2b-PB). Arrowhead (<): 48 kDa Ada2b-isoform.

To address the question if a metazoan ADA complex exists, we followed up on a report which indicated that a small Ada2b complex may be present in *Drosophila* (38). Upon re-evaluation of these purifications we noted that Ada2b was present in two peaks, consistent with the presence of Ada2b in complexes smaller than SAGA. Thus, we examined nuclear extracts for the presence of a smaller Ada2b complex. Nuclear extract was prepared from both 0–12h embryos and S2 cells that expressed C-terminally Flag-HA tagged Ada2b isoform B (Ada2b-PBH_1_F_2_). Size exclusion chromatography using Superose 6 was used to separate putative Ada2b complexes by size and shape (Figure 2A, Supplemental Figure S2). Large complexes and/or potentially aggregated proteins eluted directly after the column void volume in fraction 6+7 (Figure 2A). Ada2b peaked in fractions 9–11, which correspond to the 1.5–1.8 MD SAGA complex. As anticipated, the SAGA subunit Gcn5 co-eluted in this size range. Interestingly, a second Ada2b-containing complex was observed in fractions 17–19, corresponding to a ∼440 kDa complex if the complex behaves as the globular marker proteins. Also, in fractions 17–19 Gcn5 was again found to co-eluted with this smaller complex. This observation is consistent with the possibility that the Gcn5 HAT was associated with Ada2b in this smaller complex. Note that only the 62 kDa Ada2b isoform B could be detected in the SAGA and small Ada2b complex.

To address whether this small Ada2b complex was a consequence of perturbations in relative stoichiometric subunit abundance upon overexpression of C-terminally FLAG-HA tagged Ada2b-PB, we analysed nuclear extracts from wildtype embryos and S2 cells (Figure 2B) for the presence of this complex (Figure 2A). The gel filtration profile of endogenous Ada2b from embryos and S2 cells was comparable to that described for the tagged protein above and showed again the presence of a small nuclear Ada2b complex. These experiments suggest that a distinct small Ada2b complex is present in the nuclei of wild-type cells and flies and its presence was not driven by Ada2b overexpression.

Next, we considered the possibility that the small Ada2b complex might have resulted from dissociation of the larger SAGA complex during extract preparation or gel filtration. To address whether nuclear extraction with 420 mM high-salt buffer disturbed critical ionic interactions, we prepared extracts with 150 mM salt and analysed Ada2b complexes by gel filtration. The small Ada2b complex was still detected, which indicates that it does not form due to salt-induced destabilization of SAGA (Supplemental Figure S3).

**Figure 3.**
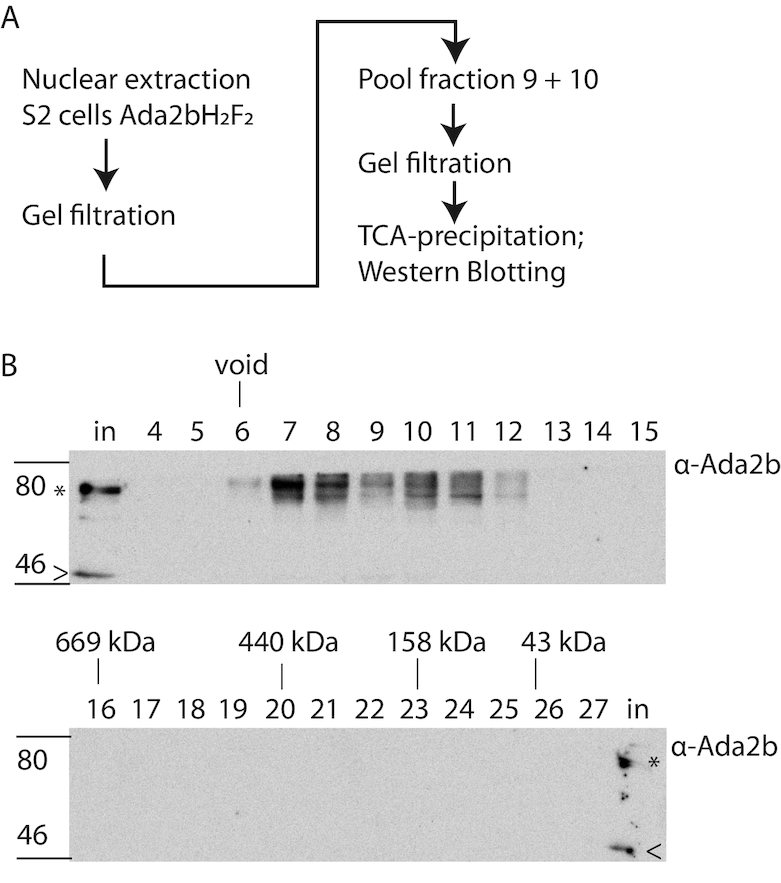
The small Ada2B-containing complex is not a consequence of SAGA disassociation during purification. (**A**) Workflow for sequential size exclusion fractionation of high molecular weight nuclear protein complexes. Nuclear extract (1.5 mg) from S2 cells was size fractionated by Superose 6 gel filtration (Figure 2, A lower panel). Fractions 9 and 10 were pooled and subjected to a second round of gel filtration. The resulting fractions were TCA-precipitated, and the gel filtration profile examined by Western blot. (**B**) Gel filtration profile of Ada2b samples subjected to re-fractionation. Ada2B was not detected in fractions 17–19, indicating that the smaller complex is not a consequence of SAGA dissociation during gel filtration. Note that the high molecular weight peak is broader upon repeat fractionation (fractions 6–11 versus 9 and 10) suggesting that some aggregation and disassociation do occur. Fraction numbers are indicated, In, input. Asterisk (*): 62 kDa Ada2b-isoform B (Ada2b-PB). Arrowhead (<): 48 kDa Ada2b-isoform.

Lastly, we addressed whether the SAGA complex disassociated during gel filtration. To test this, nuclear extracts were subjected to gel filtration and fractions containing the intact SAGA complex were recovered and subjected to a second round of gel filtration (Figure 3A). We reasoned that if dissociation of SAGA during gel filtration chromatography gave rise to the small Ada2b complex, we would again detect this smaller complex. However, no small 440 kDa Ada2b complex was observed upon repeated fractionation (Figure 3B), indicating that the SAGA complex is stable during gel filtration under these conditions. To ensure that Ada2b protein levels were above the detection limit, a second analysis was performed with a four-fold increase in nuclear extract. This was particularly important since the input material for the re-run is only ∼8% the amount of total protein used as input to recover the SAGA complex from nuclear extract in the first gel filtration column (Supplemental Figure S4). A small Ada2b complex was still not detected.

These findings together indicate that nuclear Ada2b from both fly embryos and S2 cells is part of a novel stable endogenous nuclear protein complex of ∼440 kDa and is not simply a consequence of SAGA complex dissociation during purification. We refer to it hereafter as the ADA complex.

### The *Drosophila* ADA complex is composed of Gcn5, Ada2b, Ada3 and Sgf29

**Figure 4.**
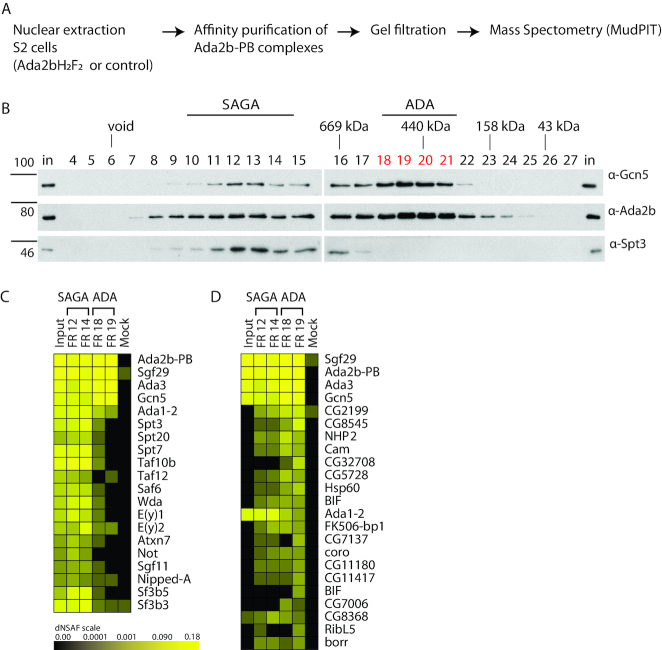
Affinity purification of the small ADA complex from S2 cell nuclear extract for MudPIT analysis. (**A**) Workflow. Ada2b-PB complexes were affinity purified from nuclear extract of S2 cells expressing Ada2b-PBH_2_F_2_ and separated by gel filtration. Mock affinity purifications were performed on nuclear extract from cells that do not express a Flag-tagged protein. Select samples were subjected to MudPIT analysis. (**B**), Flag affinity-purified Ada2b-PB complexes were subjected to gel filtration by Superose 6, and samples of each fraction were probed for Gcn5, Ada2b and Spt3 by Western blot. As in fractionated crude nuclear extract, two Ada2b peaks were observed. The SAGA complex eluted in fractions 10–14. The small ADA complex eluted in fractions 18–21. Unfractionated affinity-purified Ada2b complexes (‘input’), SAGA fractions 12 and 14, ADA fractions 18 and 19, and mock purification samples were subjected to MudPIT. (**C** and **D**) Heatmaps show relative protein abundance expressed as dNSAF (distributed normalized spectral abundance factor) with the brightest yellow indicating high abundance and decreasing intensity reflecting progressively lower abundance. The abundance of various SAGA subunits in the affinity-purified FLAG elution input for gel filtration, gel filtration fractions 12 and 14, gel filtration fractions 18 and 19, and averaged mock samples are presented as columns. Individual proteins are as indicated in each row. (C) The abundance of SAGA subunits in the high molecular weight fractions compared to those in the smaller ADA complex, along with input and mock controls. Subunit composition of the SAGA complex fractions show that Ada2b purification pulls down known SAGA subunits, indicating that the passage over the gel filtration column does not affects the detection of the subunits. (D) Non- SAGA subunits that uniquely co-purify with the ADA complex. The dNSAF values for fraction 18 and 19 were averaged and sorted in decreasing order. Proteins also enriched in the mock sample were omitted. No additional proteins co-purified uniquely with Ada2b in the ADA complex. Only Sgf29, Gcn5 and Ada3 are strongly enriched in this complex. Other proteins are either 1) not specific to the ADA complex and copurify with SAGA 2) have low dNSAF values in the ADA complex and/or 3) are common contaminants.

To study the composition of the fly ADA complex, it was affinity-purified from S2 cells expressing HA_2_ Flag_2_ tagged Ada2b isoform B, hereafter abbreviated as Ada2bH_2_F_2_ (Figure 4A). The affinity-purified proteins were resolved on a silver-stained gel and showed enrichment of proteins that corresponded with the molecular masses of known SAGA subunits (Supplemental Figure S5). Importantly, several protein bands were readily detected in the Ada2b affinity purified sample compared to the mock control. Flag affinity purified Ada2bH_2_F_2_ and associated proteins were separated by gel filtration as above, and fractions were analysed for the presence of Ada2bH_2_F_2_ by Western blot. Ada2b-PB-containing complexes migrate over a wide range, indicative of complexes of different size and shape B (Figure 4C). Two Ada2b-PB peaks were observed (Figure 4C fractions 11–15 and 19–20), both also containing Gcn5 and likely corresponding to SAGA and ADA, respectively.

Fractions 12+14 (SAGA) and 18+19 (ADA) were chosen for analysis by Multidimensional Protein Identification Technology (MudPIT) along with the affinity purified sample prior to gel filtration (input: In). As shown in Supplemental Table S1 and Figure 4C, all SAGA subunits were enriched in fraction 12+14, consistent with its identification as SAGA based on its apparent size during gel filtration. Of proteins that co-purified with Ada2bH_2_F_2_ in the ADA fractions, only the SAGA HAT-module subunits Ada2b, Gcn5, Ada3 and Sgf29 were significantly enriched (dNSAF greater than 0.035) (Supplemental Table S1, Figure 4C, D).

Of note, in addition to the lack of other SAGA subunits in fraction 18, no other novel proteins consistently and uniquely copurified with Ada2b in the ADA complex and not SAGA complex or mock (Supplemental Table S1, Figure 4D). This finding is particularly relevant since the ADA complex in yeast also contains Ahc1 and Ahc2. However, since Ahc1 and Ahc2 orthologs were not identified by protein BLAST and therefore do not appear to be present in *Drosophila*, it is not surprising that the proteomic analysis did not detect orthologs of Ahc1 and Ahc2 in the ADA fractions.

The summed molecular weights of the metazoan ADA complex subunits Ada2b-PB, Gcn5, Ada3 and Sgf29 account for 249 kDa, assuming a 1:1:1:1 stoichiometry. This size clearly differs from the apparent size by size exclusion chromatography (∼440 kDa). A stable dimer of the tetrameric ADA complex (2 × ∼ 250 kDa) could account for this size difference. We do note a doublet upon probing for Ada2b-PB (Figure 4B, fractions 18–21) that, in principle, could represent untagged 62 kDa Ada2b-PB in a dimeric complex with tagged 64 kDa Ada2b-PB. However, this band is observed in a cell line containing no tagged Ada2B (Figure 2A, Figure S2 cells and Supplemental Figure S6A, mock input), suggesting that it is nonspecific. In an attempt to test for a dimeric complex, we tried to co-IP endogenous Ada2b-PB with Ada2b-PBH_2_F_2_ (Supplemental Figure S6A). However, these results were inconclusive. Thus, these data neither eliminates nor supports the hypothesis that the discrepancy in the size of the ADA complex is due to it existing as a dimer.

It is also possible that the discrepancy in calculated size of the ADA complex versus size observed by gel filtration is due to an elongated shape. We therefore determined its molecular weight. Siegel and Monte's Svedberg formula was applied to calculate the weight of protein complexes based on hydrodynamic properties of proteins (see Supplemental Material and Methods) (45). The Stokes radius was determined for the HAT subcomplex and found to be 7.8 nm based on its elution time relative to the markers on a Superose 6 30/1000 column (Supplemental Figure S6B, C). In density centrifugation, the ADA complex was present in the fraction that roughly corresponded to 7.3 S when counting fraction 19 as peak for the ADA complex S (Supplemental Figure S6D). This results in a calculated mass of the ADA complex of 239 ± 20 kDa. This result supports the possibility that the ADA complex has an elongated shape. Our findings are consistent with findings for a recombinant yeast ADA complex, which had a gel filtration estimated mass of 434 ± 64-kDa but had a calculated mass of 180 kDa for Gcn5-Ada2-Ada3 (21). Thus, the ADA complexes appears to have an elongated shape.

In summary, the *Drosophila* Ada2b complex, like the yeast ADA complex, contains Gcn5, Ada2b, Ada3 and Sgf29, but no other unique subunits. It is similar, but not identical to the yeast ADA complex.

### Histone acetylation transferase activity of the *Drosophila* ADA complex

**Figure 5.**
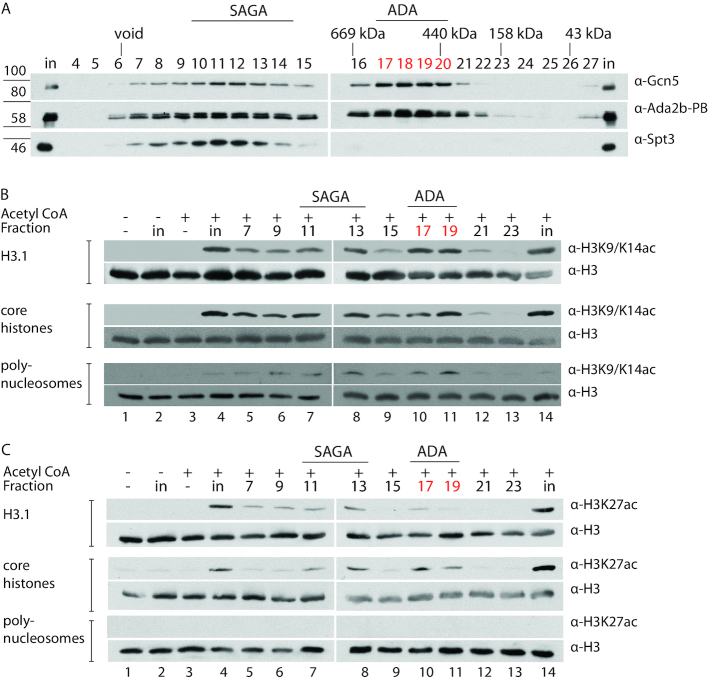
The Drosophila ADA complex has histone acetyltransferase activity *in vitro*. Ada2b-PB complexes were affinity purified overnight from nuclear extract of S2 cells expressing Ada2b-PBH_2_F_2_ and separated by gel filtration. (**A**) The gel filtration profile of Gcn5, Ada2b and Spt3. The SAGA complex eluted in fractions 10–14, and the ADA complex in fractions 17–20. B, H3K9/14 HAT assays using fractions from A that correspond to the Gcn5-containing SAGA and ADA complexes. Column fractions of affinity-purified Ada2b-PB were tested for HAT activity on recombinant human histone 3.1 (top two panels), HeLa cell core histones (middle two panels), and HeLa cell polynucleosomes (bottom two panels). Negative controls included histone substrate only, to control for background histone acetylation (lane 1), input without Acetyl-CoA to control for spurious HAT activity (lane 2), and Acetyl-CoA without HAT-containing extract to control for nonspecific acetylation due to excess substrate and cofactor (lane 3). Lane 4 includes a positive control in which the HAT assay was performed with the gel filtration input prior to size-separation of the Ada2b-PB-containing complexes. For all histone substrates, peaks of H3K9/H3K14 acetyltransferase activity were observed in fraction 13, coinciding with the SAGA complex, and fractions 17 and 19, coinciding with the ADA complex. (**C**) H3K27 HAT assays using Gcn5-containing SAGA and ADA complexes. Column fractions of affinity-purified Ada2b-PB were tested for HAT activity with the substrates and controls described in (**B**) above. No H3K27 acetylation was observed for the H3.1 and core histone controls (lanes 1–3), indicating that the antibody is specific, and does not detect H3K27ac that is naturally present on acid-extracted core histones. H3K27 acetylation is highest in the SAGA and ADA-containing fractions, correlating with the amount of Gcn5. In contrast, neither the ADA complex (lanes 10 and 11) nor SAGA complex (lane 7 and 8) acetylate lysine 27 on polynucleosomes.

The yeast ADA complex is a bona-fine HAT complex that acetylates core histones and nucleosomal substrates (7,10). We tested if the *Drosophila* ADA complex had a similar activity. To do so, we affinity-purified Ada2b-PB complexes from tagged S2 cells and separated the complexes by gel filtration. As in nuclear extract (Figure 2), Gcn5 and Ada2b eluted the fractions directly after the void volume (Figure 5A). To now better define the SAGA complex, we also probed the gel filtration profile of Spt3, a subunit of the structural SAGA SPT module. The overlap of Spt3, Gcn5 and Ada2b indicates that the SAGA complex eluted mostly in fractions 9–14, consistent with the purifications described in Figure 4B. The ADA complex eluted in fractions 17–19, also consistent with the data presented in Figures 2 and 4 (Figure 5A).

As Gcn5 is the acetyltransferase subunit of both the SAGA and ADA complexes, we tested the acetylation activity of the ADA complex-containing fractions relative to that of other fractions with a similar or higher Gcn5 content. HAT assays were performed using alternate gel filtration fractions from 7 to 23 of affinity-purified Ada2b-PB (Figure 5B) that were incubated with different histone substrates and Acetyl-CoA, and then probed for acetylation by Western blots.

Previous studies indicated that recombinant Gcn5 has robust activity on recombinant histone residues H3K9 and H3K14 (26). Therefore, we first tested if Gcn5, as part of the ADA complex, retained any activity on recombinant H3.1 at these lysine residues (Figure 5B; top two panels). H3.1 acetylation peaked in fractions 7–13, which correspond to the SAGA complex. H3.1 acetylation peaked again in 17 and 19, which correspond to the ADA complex. These results indicate that the ADA complex has HAT activity comparable to that of the SAGA complex on K9 and K14 of recombinant H3.1.

Next, HAT activity was tested on core histones (Figure 5B; middle two panels). It has been shown that the yeast SAGA complex acetylates core histone substrates from HeLa cells more efficiently than recombinant histone substrates (7). One explanation is that acid-extracted HeLa core histones retain endogenous histone marks that help the recruitment and/or add to the enzymatic activity (44). We tested whether HeLa cell core histones also serve as a substrate for the HAT activity of the ADA complex. Gel filtration fractions of affinity- purified Ada2b-PB were examined for H3K9Ac/K14Ac on acid-extracted HeLa core histones. As expected, the SAGA complex acetylated core histones at H3K9/K14 (lane 5–8 in Figure 5B; middle two lanes). The ADA complex also acetylated core histones (lane 10+11 in Figure 5B; middle two lanes).

It has been shown that recombinant Gcn5P cannot acetylate histones within nucleosomes, yet the yeast SAGA and ADA complexes can (26). To test if this behaviour is conserved in Drosophila, ADA HAT activity was tested using polynucleosomes as a substrate (Figure 5B; bottom two panels). Both the SAGA (lanes 6–8) and ADA complexes (lanes 10+11) showed a significant increase in acetylation over other fractions, indicating that the ADA complex acetylates both nucleosomal substrates and isolated (core) histones.

Finally, we tested if H3K27, which is not a preferred target of Gcn5, was acetylated by the ADA complex (Figure 5C) (46,47). H3K27 was a substrate for both ADA and SAGA when in recombinant H3 or when in acid-extracted HeLa core histones (Figure 5C. Top and middle lanes). No H3K27 acetylation was observed for the H3.1 and core histone controls (lanes 1–3), indicating that the antibody is specific. The H3K27 acetylation signal increases in the SAGA and ADA-containing fractions, indicating that it correlates with the amount of Gcn5 and thus enzymatic activity. This may reflect promiscuous HAT activity, as a lot of accessible histone substrate is available in this in-vitro assay. In contrast, neither the ADA complex nor SAGA complex acetylate H3K27 in polynucleosomes (Figure 5, C bottom two panels). Thus, nucleosomal substrates show a more restricted acetylation pattern that depends on the full complex. Taken together, this data shows that the metazoan ADA complex is a bona fide HAT complex.

## DISCUSSION

### Characterization of a metazoan ADA complex

The present study characterizes a metazoan ADA complex. It is composed of Gcn5, Ada2b isoform B, Ada3, and Sgf29. We show that this complex does not result from disassociation and ionic destabilization of the large SAGA complex, but rather exists under natural conditions within the nucleus. ChIP-Seq data suggests that the ADA complex has the capacity to bind chromatin *in vivo*. We report that the ADA complex displays nucleosomal HAT activit*y in vitr*o. Thus, in addition to its function as part of the SAGA complex, the tetrameric Gcn5 HAT module exists as an independent acetyltransferase complex.

The ADA complex in yeast contains two unique subunits, Ahc1 and Ahc2 (27,28). We did not observe these or other unique subunits in the fly ADA complex, which is consistent with the fact that there are no orthologs for Ahc1 and Ahc2 in the *Drosophila* genome. Deletion of yeast Ahc1 results in the specific loss of the ADA complex from chromatographic profiles suggesting it is required for the integrity of the complex (27). By contrast, the *Drosophila* ADA complex does not appear to require any subunits beyond the canonical HAT module for its stability. Ahc2 was recently picked up in a screen for proteins that would function as transcriptional activators when fused to a Gal4 DNA-binding domain (48) and shown to interact with autonomously replicating sequence-binding factor 1 (Abf1), a DNA-binding protein involved transcriptional regulation and chromatin reorganisation among other processes (49). Abf1 plays a key role in asymmetrically distribution of nucleosome around the HML-I or HMR-E silent mating type cassettes and is important for appropriate silencing of these regions (50). This function might point to a yeast-specific role of Ahc1/2 that is not conserved in higher eukaryotes and might explain how a stable and active ADA complex may exist in metazoans in the absence of these subunits. There are no phenotypes that are attributed to loss of Ahc1 or Ahc2 in yeast that are also attributed to loss of SAGA subunits or other GNAT complexes. This suggest that the ADA complex does serve distinct functions.

By ChIP-Seq we have identified a number of Ada2b peaks in the *Drosophila* genome, which are not apparently occupied by another SAGA subunit Spt3 (Figure 1). These may represent binding sites of the ADA complex and like those of SAGA are centered around the transcription start sites of genes. However, the ADA complex lacks the SAGA subunit TRRAP/Tra1, which is thought to be the primary target for DNA-binding transcription factors to recruit SAGA to specific genes (51,52). While the ADA complex lacks TRRAP/Tra1, it does contain additional protein domains which could facilitate its recruitment to promoters. These include the Ada2b SANT domain, a putative DNA binding domain (53) and the tandem tudor domains of Sgf29, which bind histone H3 methylated on lysine 4 (54), a well-established mark of active promoters (55).

### Functional submodules of the SAGA complex

Sharing subunits and submodules is a common theme amongst large chromatin remodelling complexes and co-activator complexes. It has been proposed that these shared components are a consequence of evolution from a common ancestor complex. Sharing subunits may be a mechanism for coordinating the activities of distinct complexes, may orchestrate the recruitment of distinct complexes to specific loci, or may simply have evolved to promote efficiency and share a common tool (56). The ADA complex subunits are incorporated in larger megadalton-sized GNAT-type HAT complexes in yeast and higher eukaryotes such as the SAGA and SAGA-like complexes (11,17,31,57,58). However, this and other recent studies have indicated that enzymatic subunits of the SAGA complex may function as independent histone modifying complexes. In this study through a combination of biochemistry and genomics, we have shown that the HAT module of the SAGA complex functions independently as the ADA acetyltransferase complex. Studies on the deubiquitinase (DUB) module of the SAGA complex have shown that it can also function independently of SAGA in the nucleus (39) and compete with other deubiquitinases for associated subunits in the nucleus and the cytoplasm (59,60). Indeed, it appears that the SAGA complex itself is essentially a coactivator complex that has sequestered the enzymatic modules necessary for appropriate signaling at promoters during gene activation.

## DATA AVAILABILITY

Original data underlying this manuscript can be accessed from the Stowers Original Data Repository at http://www.stowers.org/research/publications/LIBPB-1323_2018

Mass spectrometry data has been deposited to ProteomeXchange accession: PXD011770 http://proteomecentral.proteomexchange.org/cgi/GetDataset?ID=PXD011770

## Supplementary Material

Supplementary Data
