# Supplementary material for: Characterization of a metazoan ADA acetyltransferase complex
Source: Nucleic Acids Res. 2019 Jan 31;47(7):3383–94. doi: 10.1093/nar/gkz042 (PMC6468242; doi:10.1093/nar/gkz042)
Supplement: Supplementary Data [file gkz042_supplemental_files.zip › Characterization of a metazoan ADA acetyltransferase complex_Soffers et al 2018_revised_12248018_supplementalTables_legends.docx]

Supplemental Table 1. Subunits of the *Drosophila* ADA complex**.** The table summarizes proteins that were identified by MudPIT in two replicate purifications of the ADA complex. Average distributed normalized spectral abundance factor (dNSAF) values are listed. Proteins that co-purified with Ada2b-PB Ada2b-H_2_F_2_ in the Flag eluate (“input”), SAGA-containing fraction 14, and ADA-containing fraction 18 were analyzed by MudPIT. Proteins were sorted for average dNSAF values in the Ada2b-PB Flag purification “input” column. Proteins that were detected in only one replicate of the ADA complex-containing fraction were omitted, unless they were known SAGA subunits. Subunits Gcn5, Ada3, and Sgf29 co-purify with Ada2b-PB Ada2b-H_2_F_2_ in ADA complex-containing fraction 18. All SAGA subunits co-purified with Ada2b-H_2_F_2_ in the SAGA-containing fraction, indicating that the method is sufficient to quantitively detect complex subunits after gel filtration, including subunits known to be in low abundance in purifications such as Nipped-A or Nonstop. The spectral counts of four negative control samples (input and gel filtration fractions 6, 14, and 18) were merged. Contaminants were removed if the dNSAF exceeded 0.001 in the merged mock samples.

Supplemental Table 2. Proteins identified by MudPIT analyses of FLAG-affinity purified Ada2BH_2_F_2_ vs. Merged FLAG-Controls.
