# Supplementary material for: Characterization of a metazoan ADA acetyltransferase complex
Source: Nucleic Acids Res. 2019 Jan 31;47(7):3383–94. doi: 10.1093/nar/gkz042 (PMC6468242; doi:10.1093/nar/gkz042)
Supplement: Supplementary Data [file gkz042_supplemental_files.zip › Supplemental Materials and Methods_Soffers et al. 2018.docx]

1. **Supplemental materials and methods:**

*Ada2b dimer test*. Nuclear extract was prepared from 1 e^8^ wild type S2 cells and the stable S2 cell line expressing Ada2b-PBH_2_F_2_ as described for the preparation of crude S2 nuclear extract in this publication. The salt concentration was gradually adjusted to 150 mM NaCl by dilution with Dignam buffer (10mM HEPES pH 7.5, 10% glycerol, 10mM KCl, 1.5mM MgCl_2_, 1 mM PMSF, 0.2% [wt/vol] leupeptin, 0.2% [wt/vol] pepstatin A, and 1x Roche cOmplete Protease Inhibitor Cocktail Tablets). Extracts were treated with 25 U benzonase (Millipore Sigma, Burlington, MA, USA) per 10 mg for 10 minutes on ice and centrifuged for 15 minutes at 15 000 x g. The resulting cleared lysate (1.3 mg protein) was incubated overnight with 20 μl magnetic anti-FLAG beads (Pierce Anti DYKDDDDK Magnetic Agarose, Thermo Scientific) at 4 °C with rotation. The next day the lysate was removed (“unbound” fraction) and stored, and the beads were washed four times for ten minutes at 4 °C with rotation with 20mM HEPES pH 7.5, 10% glycerol, 0.35M NaCl, 1mM MgCl_2_, 0.1% Triton X-100. The beads were resuspended in 20 ul 2x loading dye, boiled for 10 minutes at 95 °C and vigorously vortexed. For the Western blot, 10 ug of the input sample was loaded and an equivalent volume of the diluted unbound sample, and 5 % of the eluates.

*Stokers radius calculation*. The Stokes radius (Rs) of the Ada complex was calculated with the standard curve the logarithm of the Stokes radius versus the partition coefficient Kav for the marker proteins (57). Kav equals (Ve-Vo)/(Vt-Vo), where Ve is elution volume, Vo is the void volume (7.4 mL), Vt is the total volume of the column (24mL) and the Log(Rs).

*Density centrifugation.* Affinity-purified Ada2b-PB complexes from 50 mg of nuclear extract from 0-12h Ada2b-PBH_1_F_2_ embryos in 100 μL elution buffer (1.0 mg/mL 3xFlag in 150mM NaCl, 1.5mM MgCl2, 5% glycerol, 0.05% Triton X-100, 20mM HEPES pH 7.5, 1 mM PMSF, 0.2% [wt/vol] leupeptin, 0.2% [wt/vol] pepstatin A, and 1x Roche cOmplete Protease Inhibitor Cocktail Tablets) were applied to a four mL 15-35% glycerol gradient in elution buffer. The diffusion coefficient was determined after centrifugation at 4 °C at 50 000 rpm for six hours at 4 °C. The 150 μL fractions were TCA-precipitated, dissolved in 6M urea and boiled in 6x loading dye followed by detection of Ada2b by Western blotting. The standard curve was generated for the 4-mL gradient of 50 μg thyroglobulin (19S) and 50 μg carbonic anhydrase (~2.8 S) in 100 μL elution buffer. Per fraction, 10 μL was used to detect the markers by Coomassie staining. The linearity of the gradient was checked by refractometry on a mock gradient run with an equal volume of elution buffer. The diffusion coefficient was extrapolated from the standard curve.

An approximation of the molecular weight of the Ada complex was determined by the Svedberg equation (equation 1) to obtain M (mass) as a function of the Stokes radius (R) and sedimentation coefficient (S) (58)

*S* = *M*(1 − *v*_2_ρ)/*N_o_f* = *M*(1 − *v*_2_ρ)/(*N_o_*6πη*R_s_*) eq. 1

*M* is the mass of the protein molecule in Dalton; *N*_o_ is Avogadro's number, 6.023 × 10^23^; *v*_2_ is the partial specific volume of the protein; typical value is 0.73 cm^3^/g; *ρ* is the density of solvent (1.0 g/cm^3^ for H_2_O); *η* is the viscosity of the solvent (0.01 g/cm^-s^ for H_2_O).

Setting *η to 0.01, v*_2_ρ to 0.73m S is in Svedberg units and R _s_ to nanometer, we can simplify further

M=SN_o_ (6πηR_s_)/(1−v_2_ ρ) M=SN_o_(6πηR_s_)/(1−v2_ρ_) eq. 2

M=4,205(SR s)  eq. 3

*Mass spectrometry:* All MS/MS datasets were searched against the most up‐to‐date *Drosophila melanogaster* protein sequences downloaded from NCBI (released on 2013‐02‐20). The result files from the ProLuCID search engine were processed with DTASelect (v 1.9) to assemble peptide level information into protein level information. Our in‐house software, swallow (v 0.0.1), worked with DTASelect to select Peptide Spectrum Matches such as the FDRs at the peptide and protein levels were less than 5%.

The detected peptides and proteins were compared using CONTRAST. Combining all runs, proteins had to be detected by at least 2 peptides. Proteins that were subsets of others were removed using the parsimony option in DTASelect on the proteins detected after merging all runs. Proteins that were identified by the same set of peptides (including at least one peptide unique to such protein group to distinguish between isoforms) were grouped together, and one accession number was arbitrarily considered as representative of each protein group.

Our in‐house quantitative software, NSAF7 (v 0.0.1), was used to create the quantitative Contrast

Report on all detected peptides and non‐redundant proteins identified between runs.
